# The young and the hesitant

**DOI:** 10.1016/j.lanwpc.2021.100294

**Published:** 2021-09-27

The COVID-19 pandemic has ravaged many health-care systems worldwide. For much of the pandemic, the outbreak has been approached with a so-called zero COVID-19 policy in several Western Pacific countries, including Australia, New Zealand, and Singapore. This strategy successfully contained and reduced the effect on communities when the SARS-CoV-2 alpha (B.1.1.7) variant was the dominant threat. However, as viruses do, mutations have emerged with varying degrees of danger to the population. WHO has categorised some of these mutations as variants of interest, indicating the emerging risk they pose. When these variants affect global public health with either increased transmissibility, virulence, or capacity to reduce the effectiveness of current protection measures, they are termed variants of concern.

The most prominent variant of concern in the Western Pacific region now is the delta (B.1.617.2) variant. This variant has produced a shift in the epidemiology of COVID-19. A study in *The Lancet Infectious Diseases* has shown that patients infected with the delta variant faced more than two times the risk of hospital admission and were more likely to require hospital attendance during their illness than those infected with the alpha variant. Furthermore, people afflicted with the new variant tended to be younger and unvaccinated.

The increased virulence and transmissibility of the delta variant being spread by a younger and more mobile population has enabled it to evade the current containment measures. The delta variant has brought an end to the success of zero COVID-19 strategies in many countries. Millions have been sent back into lockdown, workplaces and schools closed, and the already overstretched health-care systems in many countries, such as Australia and the Philippines, have had to cope with an even greater burden. Countries that pursued zero COVID-19 have now realised that they need to plan for living with COVID-19 as the virus will not simply disappear. For this approach to work, a high vaccination coverage is crucial. Thus, the public health message has only grown clearer: vaccination is the way forward.

A substantial challenge to increasing vaccine coverage has been hesitancy. In this issue of *The Lancet Regional Health – Western Pacific*, two studies assess the extent of vaccine hesitancy in vastly different populations. Shuhei Nomura and colleagues evaluated the reasons for vaccine reluctance in Japan. Vaccine confidence in this east Asian country is one of the lowest in the world. The authors identified that up to 43•9% of their participants were unsure or entirely averse to receiving the COVID-19 vaccination. Greater hesitancy was observed in young people, women and less medically susceptible groups. At the other end of the region in New Zealand, a study investigating hesitancy in a diverse population by Kate Prickett and colleagues showed similar results. Although 70% of the participants were likely to have the vaccine when available, a sizable proportion was hesitant. Once again, the demographic of the vaccine averse were more likely to be young and female.

With the young more likely to be vaccine-hesitant while also more likely to contract to the delta variant, the goal of controlling the COVID-19 health emergency while attaining great vaccine coverage is threatened. The vaccine-hesitant should not be dismissed but instead targeted as a susceptible group to improve vaccine coverage. The study by Nomura and colleagues delved deeper into understanding why people were unsure and unwilling to be vaccinated and went further to identify the levels of trust the participants had in different media channels. However, we need to note that these studies have been conducted in high-income countries, and trustworthy sources of information are likely to vary in other populations. We need more of these studies in the many low-income and middle-income countries of the Western Pacific region living with the ramifications of COVID-19 to provide the building blocks for the road forward. In the meantime, studies that have investigated attitudes towards other vaccines in these countries could serve as a helpful proxy to tackle the issue of hesitancy.

These studies that aim to understand, rather than quantify, vaccine hesitancy provide policy makers with the ingredients to create a targeted approach to improve vaccine coverage. Reaching the uninformed or scared to address their concerns in ways they trust will help them make decisions based on science. Reaching these individuals means more people will be protected from the devastating health impacts of COVID-19. Reaching these individuals means greater vaccine coverage can be attained, and we can start to recover from the past 20 months of hardship.

*The Lancet Regional Health – Western Pacific*

